# PalmPred: An SVM Based Palmitoylation Prediction Method Using Sequence Profile Information

**DOI:** 10.1371/journal.pone.0089246

**Published:** 2014-02-19

**Authors:** Bandana Kumari, Ravindra Kumar, Manish Kumar

**Affiliations:** Department of Biophysics, University of Delhi South Campus, New Delhi, India; CSIR-Institute of Microbial Technology, India

## Abstract

Protein palmitoylation is the covalent attachment of the 16-carbon fatty acid palmitate to a cysteine residue. It is the most common acylation of protein and occurs only in eukaryotes. Palmitoylation plays an important role in the regulation of protein subcellular localization, stability, translocation to lipid rafts and many other protein functions. Hence, the accurate prediction of palmitoylation site(s) can help in understanding the molecular mechanism of palmitoylation and also in designing various related experiments. Here we present a novel *in silico* predictor called ‘PalmPred’ to identify palmitoylation sites from protein sequence information using a support vector machine model. The best performance of PalmPred was obtained by incorporating sequence conservation features of peptide of window size 11 using a leave-one-out approach. It helped in achieving an accuracy of 91.98%, sensitivity of 79.23%, specificity of 94.30%, and Matthews Correlation Coefficient of 0.71. PalmPred outperformed existing palmitoylation site prediction methods – IFS-Palm and WAP-Palm on an independent dataset. Based on these measures it can be anticipated that PalmPred will be helpful in identifying candidate palmitoylation sites. All the source datasets, standalone and web-server are available at http://14.139.227.92/mkumar/palmpred/.

## Introduction

S-Palmitoylation (hereafter termed as palmitoylation) is a eukaryote specific [1], reversible post-translational protein modification, which covalently adds palmitate moiety (C16∶0) to a cysteine residue through a thioester linkage [2], [3]. It plays an important role in a number of cellular processes such as membrane-protein interaction [4], signal transduction [5], neuronal development [6], apoptosis [7], lipid raft targeting [8], [9] and subcellular localization [10]. Thus accurate identification of palmitoylation sites may provide important clues to decipher the underlying mechanism in the above-mentioned processes. Experimental techniques employing proteomics and imaging methods can be used for detection of palmitoylation sites. However time and resources required to search palmitoylation sites in the huge number of protein sequences present in different databanks, limit their usage. Due to this reason, only a small number of palmitoylation sites have been identified experimentally to date. Therefore an effective and highly accurate *in silico* prediction method can be very useful in rapid identification of candidate palmitoylation site which can be targeted for further experimental verification.

In recent years a few computational methods have been reported to find out palmitoylation sites by using information carried in protein sequences. Zhou et al. [11] developed the first predictor CSS-Palm by adopting clustering and scoring strategy on the dataset containing 210 palmitoylation sites with Jack-Knife sensitivity of 82.16% and specificity of 83.17%. Another predictor NBA-Palm was created by Xue et al. [12] using Naive Bayes method which achieved the overall prediction accuracy of 86.74% in Jack-Knife cross-validation. Ren et al. [13] proposed version 2.0 of CSS-Palm and claimed significant improvement in performance over previous version. Wang et al. [14] added a new algorithm CKSAAP-Palm to this list which used composition of k-spaced amino acid pairs as the encoding scheme. Later Hu et al. [15] proposed another predictor, named IFS-Palm, based on the features of amino acid sequences using Nearest Neighbor Algorithm and successfully showed that the IFS-Palm achieved a significantly better performance over CKSAAP-Palm on an independent dataset. Recently one more predictor WAP-Palm [16] was reported having accuracy 85.99% and Matthews Correlation Coefficient (MCC) of 0.72 in 10 fold cross-validation.

Here we report a new support vector machine (SVM) based approach for palmitoylation site identification by using features extracted from the primary amino acid sequence information only. In order to build SVM model we extracted palmitoylated peptides of different window size and encoded the same with different input features namely sequence conservation (PSSM), secondary structure and disorder. The best result was achieved with the sequence conservation encoding on 11-mer peptide. Benchmarking results on independent datasets confirmed that the proposed method is more efficient than the recent predictors, IFS-Palm and WAP-Palm. A web-server and standalone package, termed PalmPred is also available at http://14.139.227.92/mkumar/palmpred/, to enable high throughput annotation of new palmitoylation sites.

## Materials and Methods

### Data Source

In this study, we used the dataset constructed for the development of IFS-Palm [15]. It is compiled from the Uniprot database [17] (Release: 15.9, 13-Oct-2009) by searching the keywords “Field” for ‘Sequence annotation [FT]’, “Topic” for ‘Lipidation’, “Term” for ‘Palmitoyl cysteine’, and “Confidence” for ‘Experimental’. The dataset consists of 151 proteins, which include 1537 cysteine residues in total, of which 234 residues were experimentally verified, as palmitoylation sites and remaining 1303 were not palmitoylated. The dataset was further divided into training and independent test datasets, similar to the strategy adopted in IFS-Palm.

#### Training dataset

Out of the total of 151 proteins, 132 proteins having 207 experimentally verified palmitoylated cysteines and 1140 non-palmitoylated cysteines were used as training dataset (D_train_).

#### Independent test datasets

Remaining 19 proteins having 27 experimentally verified palmitoylated cysteines and 163 non-palmitoylated cysteines were used as an independent dataset (D1_ind_).

It was clear that proteins of D1_ind_ were not present in training dataset of IFS-Palm and our method but for other predictors this may not be the case. In order to benchmark the performance of our method *vis-à-vis* other, we created another independent dataset (D2_ind_)_._ For this, we used 54 yeast proteins in which palmitoylation sites were identified and described in [18]. Eight proteins, also present in training dataset D_train_ were excluded from the D2_ind_. The resulting D2_ind_ dataset contains 46 proteins in which palmitoylation sites have been identified experimentally. This dataset was also used for independent evaluation of our method. To include any recent addition of palmitoylation sites, proteins of D2_ind_ were also searched in Uniprot from Field “Sequence annotation (FT)”, Topic “Lipidation” and Term “S-palmitoyl cysteine”.

We also compiled two more datasets for assessing the performance of our method – D3_ind_ and D4_ind_ containing 10 and 17 proteins respectively in which several palmitoylation sites were experimentally confirmed. The dataset D3_ind_ was collected from [19]. The dataset D4_ind_ was taken from [20] and consists of synaptic, motor, channels, G-protein coupled receptor, focal adhesion and tight junction proteins. We did not find any Uniprot annotation for palmitoylation in D3_ind_ and D4_ind_ proteins.

### Pattern Size for Feature Encoding

The first step of our work was to determine the optimal window length, W of the cysteine containing peptide which can give maximum performance for palmitoylation site prediction. In order to do this, we extracted peptide segments of different window sizes from each protein such that each W-mer peptide contained a cysteine, symmetrically flanked by (W-1)/2 residues. For terminal cysteine residue, where the flanking region had less than (W-1)/2 residues, appropriate number of dummy residue ‘X’ was added to complete the window.

Each peptide segment was assigned a label depending on the nature of central cysteine residue. The peptide segment having a palmitoylated central cysteine residue was labeled positive and a non-palmitoylated central cysteine residue was labeled as negative. Thus for each window we extracted a total of 207 and 27 positive labels from D_train_ and D1_ind_ respectively. Similarly the number of negative labels in D_train_ and D1_ind_ were 1140 and 163.

### Feature Encoding

#### Conservation feature

This was obtained from position-specific scoring matrix (PSSM) generated during PSI-BLAST [21] search against NR90 by three iterations of searching at e-value cut-off of 0.001 for inclusion of sequences in next iteration. The NR90 database was constructed from NR protein sequence database clustered at 90% sequence identity by using CD-HIT [22]–[24]. The PSSM contains the probability of occurrence of each type of amino acid residues at each position and hence can be considered as a measure of residue conservation at a given position. This means that evolutionary information for each amino acid is encapsulated in a vector of 20 dimensions and the size of PSSM for a protein with N residues is 20 x N. In the present work, since we were using a peptide of fixed length ‘W’ to encode a palmitoylation site, a corresponding sub-matrix of size W x 20 was extracted from each PSSM. In case of peptides containing ‘X’ (see previous section), each ‘X’ in PSSM was represented by ‘0 0 0 0 0 0 0 0 0 0 0 0 0 0 0 0 0 0 0 0′.

#### Structural disorder feature

Disordered regions are known to be rich in binding sites and provide an important locus for diverse protein post-translational modifications such as methylation and acetylation [25]. A number of studies also reported that the incorporation of structural disorder increases the prediction accuracy [26], [27]. Therefore, we also included structural disorder probability of each residue as an input feature to code the peptides. For this purpose, VSL2 predictor [28], [29] was used which assigned a score between 0 and 1 to each residue. Higher value of VSL2 score (close to 1) shows lack of fixed 3-dimensional structure while lower value shows higher propensity of fixed structure. It means larger the score is, the more likely a residue lacks fixed structure. We assigned score 0 to each dummy residue ‘X’.

#### Secondary structure feature

In their work Hu et al. [15] had reported that information of protein structure also plays an important role in the prediction of palmitoylation site. It indicates that if structural information of each amino acid can be provided into more explicit form, it may help to achieve better prediction of palmitoylation site. In the present study we provided probability of an amino acid to form each of the three secondary structures namely, helix, sheet and coil using standalone PSIPRED (Ver 3.3) [30] at default parameters. Here also NR90 was used to generate the PSSM. Similar to conservation feature, for secondary structure prediction each ‘X’ was given a hypothetical value of ′0 0 0′ to maintain uniformity with other amino acid scores.

### Support Vector Machines

We employed Support Vector Machine classifiers (SVM) to predict if, for a given input feature vector, the central cysteine residue is palmitoylated or not. SVMs, designed by Vapnik [31], are computational algorithms, which can efficiently classify complex, non-linear and high-dimensional data. So, it has been used for developing a large number of bioinformatics applications [32]–[36]. SVM trains a classifier by mapping the input vectors in higher dimension space through kernel functions and separating them into two classes (represented as positive and negative labels) with the maximal margin and least error in the transformed space. The trained classifier can be used to predict in which of the two classes an unknown sample falls, with a high confidence level. In the current study, SVM model was built using SVM-light [37] which is freely available from http://svmlight.joachims.org/. We experimented with several values of cost-factor, kernel (polynomial and radial basis function kernels) and penalty parameter C on peptides of different window sizes taken from D_train_. The model with the best performance parameters was selected as the optimal model.

### Cross-Validation

Cross-validation is a method to evaluate classifier performance. The independent dataset test, sub-sampling (k-fold cross-validation) and Jack-Knife analysis (leave-one-out) are the three popular methods for cross-validation. In k-fold cross-validation, the dataset is randomly divided into k non-overlapping sets, k-1 sets are used for training and the remaining set for testing. This process is repeated k times such that each set is used as test set once and overall performance is calculated by averaging over all test sets.

In the present study we used ‘leave-one-out’ cross-validation (LOOCV) which has been considered as the most objective method in comparison to other two methods [38]–[43]. LOOCV uses one example from dataset as testing data and the remaining as training data. In a complete cycle of LOOCV, each example is used as test. The LOOCV thus shows dynamic behavior of testing and training data where every sample is the training set to train models as well as the testing set to test model [44]. It can also exclude the memory effects that exist in the re-substitution test, and provides the unique results for a given benchmark dataset [45].

### Classifier Evaluation Measures

We adopted threshold-dependent performance matrices namely Specificity (S_p_), Sensitivity (S_n_), Accuracy (A_cc_), and Matthews Correlation Coefficient (MCC) to measure the prediction capability of our method. Sensitivity and specificity respectively are the percentage of correct predictions from positive (palmitoylated cysteines) and negative cases (non-palmitoylated cysteines). Accuracy (arithmetic mean of sensitivity and specificity) signifies the overall percentage of correctly predicted palmitoylated and non-palmitoylated peptides. The MCC [46] is a measure of predictive capability of classifiers, which reflects both the sensitivity, and specificity of the prediction algorithm. It is considered as a more reliable measure of the quality of binary classifications and can be used for unbalanced dataset also [47],[48]. The MCC value always ranges from -1 to 1. An efficient predictor will have positive correlation coefficient value. The value -1 and 0 represents opposite and random predictions respectively.

All of the above mentioned parameters can be defined as follows:
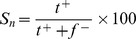





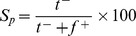





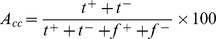





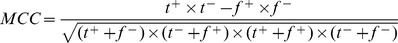



The abbreviations t^+^, t^−^, f^+^ and f^−^ represent true positive, true negative, false positive and false negative respectively. True and false positives are the predicted palmitoylated peptides, which are in reality a palmitoylated, and non-palmitoylated peptide respectively. True and false negatives are the peptides predicted as non-palmitoylated and are actually a non-palmitoylated and palmitoylated peptides respectively.

## Results and Discussion

### Performance of PSSM and Selection of Optimized Window

**Figure 1:**
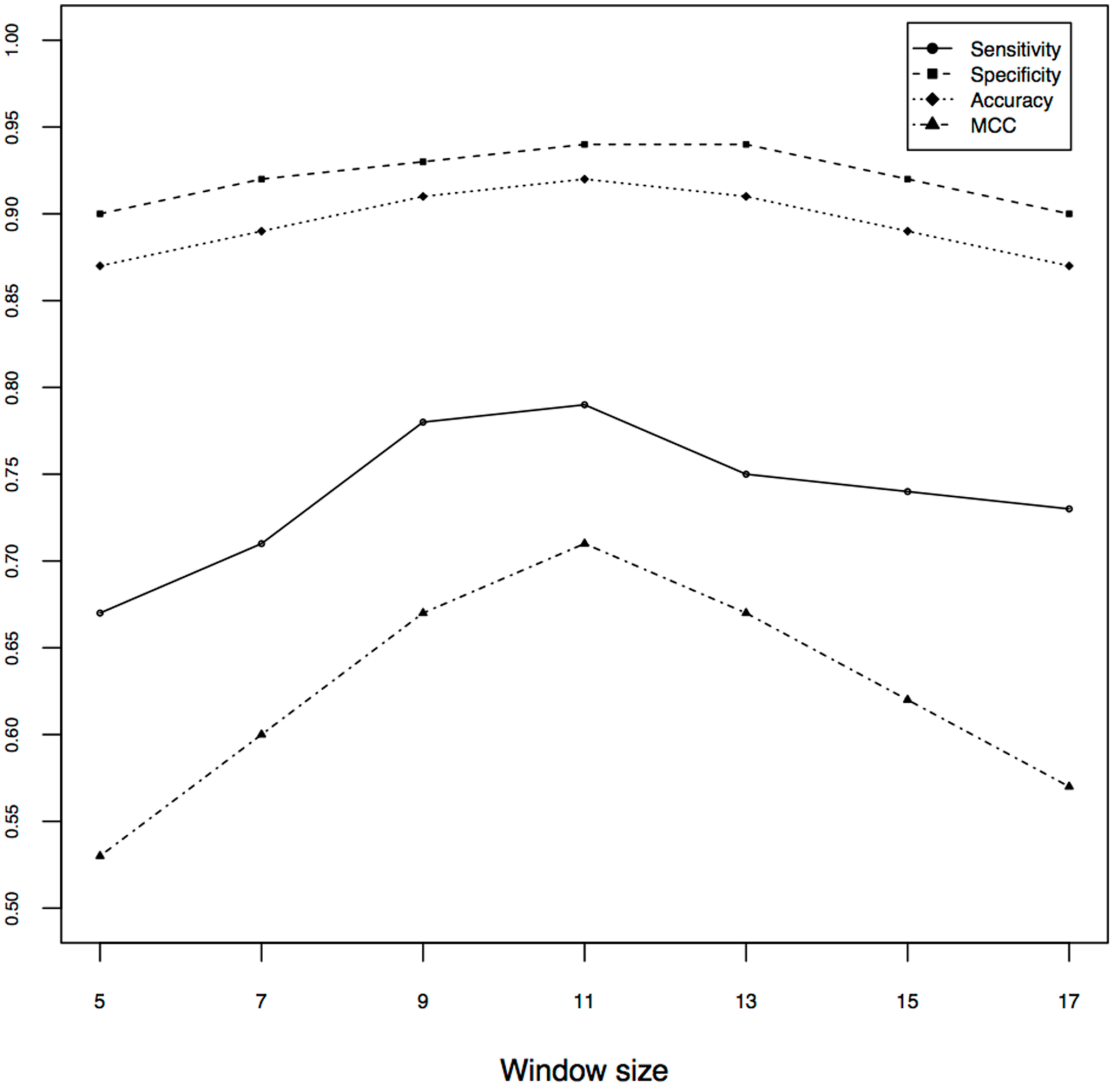
Performance of SVM on different window size.

To get optimum pattern size, we used only the evolutionary information obtained from PSSM generated by PSI-BLAST search against NR90. The performance was analyzed for window sizes 5, 7, 9, 11, 13, 15 and 17. As shown in Figure 1, the overall performance increased steadily with increase in the window-size, attained the peak at 11 and started declining afterwards. The maximum performance, which was achieved by us for pattern size 11, was 79.23% sensitivity, 94.30% specificity and 91.98% accuracy with MCC 0.71 (detailed performance in Table 1). In rest of the work, window-size 11 and PSSM based model was considered as baseline model unless mentioned otherwise. Additional features were added to the baseline model to further improve the performance.

**Table 1:** Performance of PSSM based SVM model.

Threshold	Sensitivity	Specificity	Accuracy	MCC	False Positive Rate (%) (100-specificity)
−1	94.20	36.93	45.73	0.24	63.07
−0.9	92.75	60.18	65.18	0.38	39.82
−0.8	89.37	73.77	76.17	0.47	26.23
−0.7	88.89	81.05	82.26	0.55	18.95
−0.6	85.51	86.49	86.34	0.60	13.51
−0.5	81.64	90.88	89.46	0.65	9.12
−**0.4**	**79.23**	**94.30**	**91.98**	**0.71**	**5.70**
−0.3	72.95	95.96	92.43	0.70	4.04
−0.2	67.63	96.75	92.28	0.69	3.25
−0.1	58.94	97.63	91.69	0.65	2.37
0	53.62	98.25	91.39	0.63	1.75
0.1	49.28	98.60	91.02	0.61	1.40
0.2	45.89	98.86	90.72	0.59	1.14
0.3	39.61	98.95	89.83	0.55	1.05
0.4	38.16	99.12	89.76	0.54	0.88
0.5	33.82	99.21	89.16	0.51	0.79
0.6	27.54	99.47	88.42	0.46	0.53
0.7	21.26	99.47	87.45	0.40	0.53
0.8	17.87	99.65	87.08	0.37	0.35
0.9	13.04	99.82	86.49	0.32	0.18
1	8.70	99.91	85.89	0.26	0.09

The selected performance for SVM model has been shown in bold.

### Integration of Structure Disorder Information in Sequence Profile

When we integrated the disorder scores of central cysteine and its flanking 5 amino acids (on each side) derived from VSL2, no change in performance was noticed. We obtained sensitivity of 79.23%, specificity of 94.30%, accuracy of 91.98% and MCC of 0.71, which is exactly same as the performance achieved using PSSM alone (Table 1). It is opposite to what observed by Hu et al. [15] that disordered region plays an important role in the cysteine-palmitoylation. In their work, Gao and Xu [49] had observed a very little difference in the mean disorder scores (as predicted by VSL2) for both S-palmitoylated and non-palmitoylated cysteine. This little difference between the disorder propensities may be the reason for not getting any improvement in the prediction accuracy.

### Prediction using Information in Sequence Conservation and Secondary Structure

Computing the probability score to form each of the three secondary structures by an amino acid is also a way of providing order/disorder information. Hence we also used PSIPRED predicted secondary structure information along with PSSM as input and trained the SVM. With PSSM and secondary structure information combined together, we achieved the accuracy of 91.98% and MCC of 0.71. The corresponding values of sensitivity and specificity were 79.23% and 94.30% respectively.

Again the result did not show any improvement over baseline model. This shows that addition of secondary structure information was also not able to provide any extra information to the predictor.

### Prediction using Information in Sequence Profile, Secondary Structure and Disorder

We also used a combination of both disorder and secondary structure likelihood of each residue of the peptide pattern to see the influence of both together. Contrary to our expectation we obtained no increase in accuracy of prediction. All the performance measures i.e., sensitivity, specificity, accuracy and MCC remained same as obtained with PSSM alone (Table 1).

Hence SVM model obtained with PSSM was considered the final prediction model in rest of the work and it is referred as PalmPred henceforth.

### Comparison with Existing Methods

#### Comparison of LOOCV performance

The existing methods of palmitoylation site prediction are CSS-Palm 1.0, NBA-Palm, CSS-Palm 2.0, CKSAAP-Palm, IFS-Palm and WAP-Palm. As the training data of the available predictors, except IFS-Palm, is different from the PalmPred, direct comparison among these predictors with PalmPred might not be reasonable. As described in materials and methods PalmPred and IFS-Palm has similar training dataset, so we compared the performance during LOOCV between them only. The PalmPred reached sensitivity of 79.23%, specificity of 94.30%, accuracy of 91.98% and MCC of 0.71 whereas the IFS-Palm attained sensitivity of 68.60%, specificity of 94.65%, accuracy of 90.65% and MCC of 0.64 (Table 2). The result shows that at comparable specificity, PalmPred achieved almost 10% higher sensitivity.

**Table 2:** Performance of IFS-Palm and PalmPred on training dataset (D_train_) using LOOCV approach of training.

Predictor	Sensitivity	Specificity	Accuracy	MCC
IFS-Palm	68.60	94.65	90.65	0.64
PalmPred	79.23	94.30	91.98	0.71

#### Comparison of independent dataset performance

In order to do an unbiased evaluation, it is essential to benchmark the performance on an independent dataset. We used two independent datasets namely D1_ind_ and D2_ind_ for benchmarking purpose (see materials and methods for detail).

The first dataset (D1_ind_) had a subset of 19 proteins out of total 151 proteins compiled by Hu et al. [15] for development and evaluation of IFS-Palm. The performance of CKSAAP-Palm, IFS-Palm and PalmPred was evaluated on D1_ind_. As shown in Table 3, in comparison of CKSAAP-Palm, a significant difference was observed in the performance of PalmPred. When comparison was made between IFS-Palm and PalmPred, PalmPred achieved better sensitivity though the specificity was same (Table 3). The result was consistent to the performance shown during LOOCV, where also PalmPred had achieved higher sensitivity and comparable specificity. While we were working on development of PalmPred, a new palmitoylation site prediction method, namely, WAP-Palm was published by Shi et al. [16]. As 12 out of 15 proteins constituting the independent dataset of WAP-Palm were part of PalmPred training data, we did not benchmark the performance of WAP-Palm *vis-à-vis* PalmPred.

**Table 3:** Performance of CKSAAP-Palm, IFS-Palm and PalmPred on the independent dataset (D1_ind_) of 19 proteins.

Predictors	Sensitivity	Specificity	Accuracy	MCC
CKSAAP-Palm*	62.96	86.50	83.16	0.43
IFS-Palm*	92.59	98.77	97.89	0.91
PalmPred	96.30	98.77	98.42	0.94

*The values for all measurement categories had been taken from Hu et al. 2011.

The dataset D2_ind_ was used for performance assessment of IFS-Palm, WAP-Palm and PalmPred. We took palmitoylation sites of D2_ind_ proteins predicted by IFS-Palm from [15]. As Shi et al. [16] had shown that WAP-Palm performed best at threshold 0.8 we used the same threshold for prediction. We observed that PalmPred identified 61 palmitoylation sites in 33 proteins. WAP-Palm predicted 21 palmitoylation sites in 15 proteins while IFS-Palm predicted 60 sites in 31 proteins (Table 4). When we made a comparison between PalmPred and IFS-Palm, it was observed that PalmPred predicted at least one palmitoylated site in 10 different proteins where IFS-Palm failed to predict even one site. When we compared the 24 experimentally verified palmitoylation sites by Roth et al. [18], the total number of sites predicted by WAP-Palm, IFS-Palm and PalmPred were 1, 3 and 11 respectively. For protein TLG2, Roth et al. [18] had estimated the palmitoylation at position 317 [15] but PalmPred predicted it at 316 (Table 4). We cross-checked the position in sequence of TLG2 (available at Uniprot) and found that cysteine was present at position 316. When we analyzed the prediction of PalmPred *vis-à-vis* Uniprot annotation, we observed that PalmPred predicted 29 novel sites, failed to predict 4 sites and correctly predicted 32 sites.

**Table 4:** Comparative study of cysteine palmitoylation sites in Yeast proteins. This data is referred as D2_ind_ in the text.

Protein	Uniprot ID	Uniprot annotation	Experimentally identified sites	IFS-Palm	WAP-Palm	PalmPred
TVP18	A6ZMD0	–	–	–	–	78
HIP1	P06775	–	603	339, 463	339	–
RHO2	P06781	188*	188	188	–	188
NUC1	P08466	–	–	–	–	–
TUB1	P09733	–	–	–	–	14
GPA2	P10823	4	–	4	–	4
GAP1	P19145	–	–	286	–	–
YCK1	P23291	537^#^, 538^#^	–	537, 538	–	537, 538
YCP4	P25349	243*	–	243	–	243
AGP1	P25376	633^#^	–	469	172, 266	–
SYN8	P31377	238*	238	–	–	238
MLF3	P32047	–	–	–	–	2
SSO1	P32867	–	266	–	–	266
SNC2	P33328	94*	94	94	94	94
YKT6	P36015	196^#^	–	196	–	196
YKL047W	P36090	–	–	516	–	516
BAP2	P38084	–	609	–	–	–
VAP1	P38085	–	619	318, 412	–	–
YBR016W	P38216	–	–	110, 119, 122	–	119
TAT2	P38967	–	–	489	–	–
AKR1	P39010	–	–	663	533, 667	533, 663, 667
MNN1	P39106	–	17	–	–	–
SSO2	P39926	–	270, 274	–	–	270
YCK3	P39962	517*, 518*, 519*,520*, 522*, 523*,524*	–	84, 517, 518,519, 522, 524	–	517, 518, 519,520, 522, 523
VAC8	P39968	4*, 5*, 7*	–	4, 5, 7, 106, 144	106	4, 5, 7
HEM14	P40012	–	–	104, 435	–	–
LBS6	P42951	–	–	217, 223, 531	–	217, 223
MNN11	P46985	–	35	–	–	–
MSE1	P48525	–	–	413	502	12
GNP1	P48813	–	663	193, 312	201	–
MNN10	P50108	–	44	263, 362	–	–
YGL108C	P53139	4*	–	4	–	4
RHO3	Q00245	–	5	–	130	5
MEH1	Q02205	7 *, 8*	–	7, 8	–	7, 8
TLG1	Q03322	205*, 206*	205, 206	–	–	205
YLR326W	Q06170	–	–	79, 80, 81	80	79, 80, 81
SNA4	Q07549	2*, 3*, 5*, 7*, 8*	–	–	–	2, 3, 5, 7, 34
PSR1	Q07800	9$, 10$	–	10	10	9, 10
YLR001C	Q07895	–	780	780	504	780
PSR2	Q07949	9$, 10$	–	9, 10	10	9, 10
TLG2	Q08144	**–**	317, 325	–	–	316
YPL199C	Q08954	–	–	235	–	233, 235
SAM3	Q08986	–	–	268, 321	321	–
YPL236C	Q12003	13*, 14*, 15*	–	14, 15	13, 14, 159	13, 14, 15
PIN2	Q12057	–	35, 41, 53	66, 79, 81,82, 84	66, 81, 82	53, 66, 79,81, 82, 84
VAM3	Q12241	–	262, 274	–	–	262

$ , * and # denotes the palmitoylated cysteine respectively annotated as ‘probable’, ‘By similarity’ and ‘potential’ in Uniprot.

CSS-Palm 1.0, NBA-Palm and CSS-Palm 2.0 web-servers were not functional, so we could not compare these methods.

### Database for PSSM Construction

One of the prerequisites to carry out the prediction in PalmPred is to first do the PSI-BLAST to generate input features i.e. PSSM. One major challenge in employing PSI-BLAST is that with increase in database size, PSI-BLAST search time also increases. Therefore, to speed up the PSSM generation, we used databases having less redundancy than NR90 and then evaluated the performance. For D1_ind_ proteins, we generated PSSM against NR80 and NR70 and checked their performance on the PalmPred model. NR80 and NR70 contained 80% and 70% redundancy reduced protein sequences respectively and were compiled from NCBI-NR protein sequences by using CD-HIT [22]–[24]. As shown in Table S1, with decrease in redundancy of NR database, the performance also decreased which was as reported by Ahmad and Sarai [50].

### Comparison with Other Machine Learning Classifiers

Other than SVM, several machine learning approaches have been used to develop classifiers for predicting post-translational modification sites including palmitoylation [12], [16], [51]. So besides SVM, we also tested following three machine learning methods implemented in WEKA program [52]: Naïve Bayes, RBF Network and Random forest. Similar to the SVM each of these three classifiers was constructed by incorporating PSSM score on pattern size 11. Each classifier was trained and evaluated on the training dataset (D_train_) using LOOCV. By comparing the prediction results of the Naïve Bayes, RBF Network and Random forest classifiers with SVM classifier (Table 5), it was found that SVM classifier achieved the highest specificity, accuracy and MCC. The performance on independent dataset D1_ind_ was also very poor for Naïve Bayes, RBF Network and Random forest classifiers (Table 5). The comparison clearly shows that the SVM is an ideal choice among different machine learning methods available.

**Table 5:** Performance of different machine learning classifiers.

Leave-one-out Cross-validation					Independent Testing Dataset (D1_ind_)			
Classifiers	S_n_	S_p_	A_cc_	MCC	S_n_	S_p_	A_cc_	MCC
Naïve Bayes	79.60	74.50	79.58	0.44	82.80	81.70	82.63	0.51
RBF Network	85.00	49.00	85.00	0.37	82.10	60.00	82.11	0.37
Random Forest	85.20	21.40	85.23	0.19	89.50	36.50	89.47	0.48
Support Vector Machine	79.23	94.30	91.98	0.71	96.30	98.77	98.42	0.94

S_n_, S_p_, A_cc_ and MCC represent Sensitivity, Specificity, Accuracy and Matthews Correlation Coefficient respectively.

### Web-Server

To make the optimized SVM model accessible to experimental biologists, we have developed PalmPred web-server and standalone package. The prediction output provides information about all cysteine containing peptides, the position and palmitoylation state of cysteines. The PalmPred web-server can take a maximum of 5 sequences at a time. For a query dataset of more than 5 sequences standalone version of PalmPred can be used. The PalmPred is freely available at http://14.139.227.92/mkumar/palmpred/.

### Performance Assessment of PalmPred

Recently two reports were published which experimentally established palmitoylation sites in a group of proteins. The first work was done by Nishimura and Linder [19] which experimentally identified palmitoylation sites in Rho GTPase proteins. The second work was reported by Oku et al. [20] on 17 candidate proteins predominantly expressed in brain. In order to further assess the reliability of PalmPred, we used the proteins of above-mentioned work (referred as D3_ind_ and D4_ind_ respectively in materials and methods).

Nishimura and Linder [19] reported a novel motif, CCaX, which tandomly undergoes prenylation and palmitoylation at C-terminal. In order to prove their hypothesis they worked on a set of ten proteins. They experimentally determined palmitoylation sites for five proteins and also reported a protein, PLA2γ, which is known to be palmitoylated but the site of palmitoylation present in this protein is unknown. When PalmPred was used to predict the palmitoylation site in these ten proteins, of five proteins whose palmitoylation sites were experimentally determined PalmPred could correctly determined palmitoylation sites of two of those proteins (Table 6). For PLA2γ, PalmPred predicted the candidate palmitoylation site as amino acid 539 which is consistent with the observations of [19] i.e. the predicted position lies at second C of CCaX motif. Of the remaining four proteins (PRL-1, PRL-2, PDE6α and PDE6β), whose palmitoylation sites was not determined by Nishimura and Linder, in PRL-1, PalmPred correctly predicted palmitoylation site at 171, which follows the hypothesis proposed by [19] besides one additional site at position 104 (Table 6). But in PRL-2, PalmPred predicted site did not follow the CCaX motif rule. In PDE6α and PDE6β, PalmPred did not predict any palmitoylation site which might be actually the case, as canonical CaaX processing (i.e. proteolysis and carboxymethylation after prenylation of CaaX cysteine) of PDE6α and PDE6β is well documented [53].

**Table 6:** Prediction performance of PalmPred on dataset D3_ind_ taken from Nishimura and Linder 2013 (referred as D3_ind_).

Protein	Uniprot ID	Total no. of cysteines in protein	Experimentally identified sites	PalmPred
bcdC42	P60953	7	188	–
Wrch-1	Q7L0Q8	12	256	256
RalA	P11233	3	203	–
RalB	P11234	2	203	–
PRL-1	Q93096	6	–	104, 171
PRL-2	Q12974	7	–	101
PRL-3	O75365	6	170	171
PDE6α	P16499	15	–	–
PDE6β	P23440	21	–	–
PLA2γ	Q9UP65	7	–	539

Out of the 17 proteins tested as candidate for palmitoylation, Oku et al. [20] were able to experimentally establish the palmitoylation only for 10 sites (Table 7). PalmPred was able to correctly predict 5 sites out of them. One additional site (at position Cys-3) was also confirmed by the mutational analysis in neurochondrin which was also correctly predicted by PalmPred. Among the seven proteins whose palmitoylation couldn’t be established by [20], in four proteins namely Homer 1C, Liprin-α2, Paxillin and Par3, PalmPred did not predict any palmitoylation site (Table 7). In remaining three proteins viz Kalirin7, KIF5C candidate site and palmitoylation sites were different while in one protein (Rab3A) both candidate and PalmPred predicted sites were same but no palmitoylation can be experimentally established.

**Table 7:** Prediction of PalmPred on dataset D4_ind_ taken from Oku et al. 2013.

Protein	Uniprot ID	Total no. ofcysteines in protein	PutativePalmitoylation sites	Experimentalconfirmation	PalmPred
TARPγ-2	O88602	6	121	+	68, 121
TARPγ-8	Q8VHW2	7	144	+	90, 91, 144
Cornichon-2	O35089	8	9	+	84
CaMKIIα	P11798	10	6	+	–
Kalirin7	A2CG49	55	1404	–	417, 989, 1334, 2508
Homer1C	Q9Z2Y3	2	365	–	–
Neurochondrin	Q9Z0E0	25	3,4	+	3, 4, 292, 647, 348
Rab3A	P63011	4	220	–	218, 220
Syd-1	Q9DBZ9	13	736	+	346, 360
Liprin-α2	Q8BSS9	9	3	–	–
KIF5C	P28738	10	7	–	303, 304
TRPM8	Q8R4D5	26	1032	+	780, 1028, 1031, 1032, 1033
TRPC1	Q61056	19	736	+	198, 367, 692, 703
Orexin2receptor	P58308	14	381	+	381, 382
Paxillin	Q8VI36	25	591	–	–
Zyxin	Q62523	23	404	+	–
Par3	Q99NH2	12	6	–	–

One important thing we noticed with both datasets (D3_ind_ and D4_ind_) that despite very large number of cysteines in few proteins, PalmPred predicted palmitoylation site did not increased proportionally. Rather it shows robustness and high specificity of prediction of our method. One of the possible reasons behind slightly inferior performance of PalmPred can be due to novelty of datasets on which work of [19] and [20] is based as both tried to establish palmitoylation in a new group of proteins. Even Uniprot does not have any information of palmitoylation of these proteins. We feel that with addition of new information to the database, the performance can also be improved further.

## Conclusions

**Figure 2:**
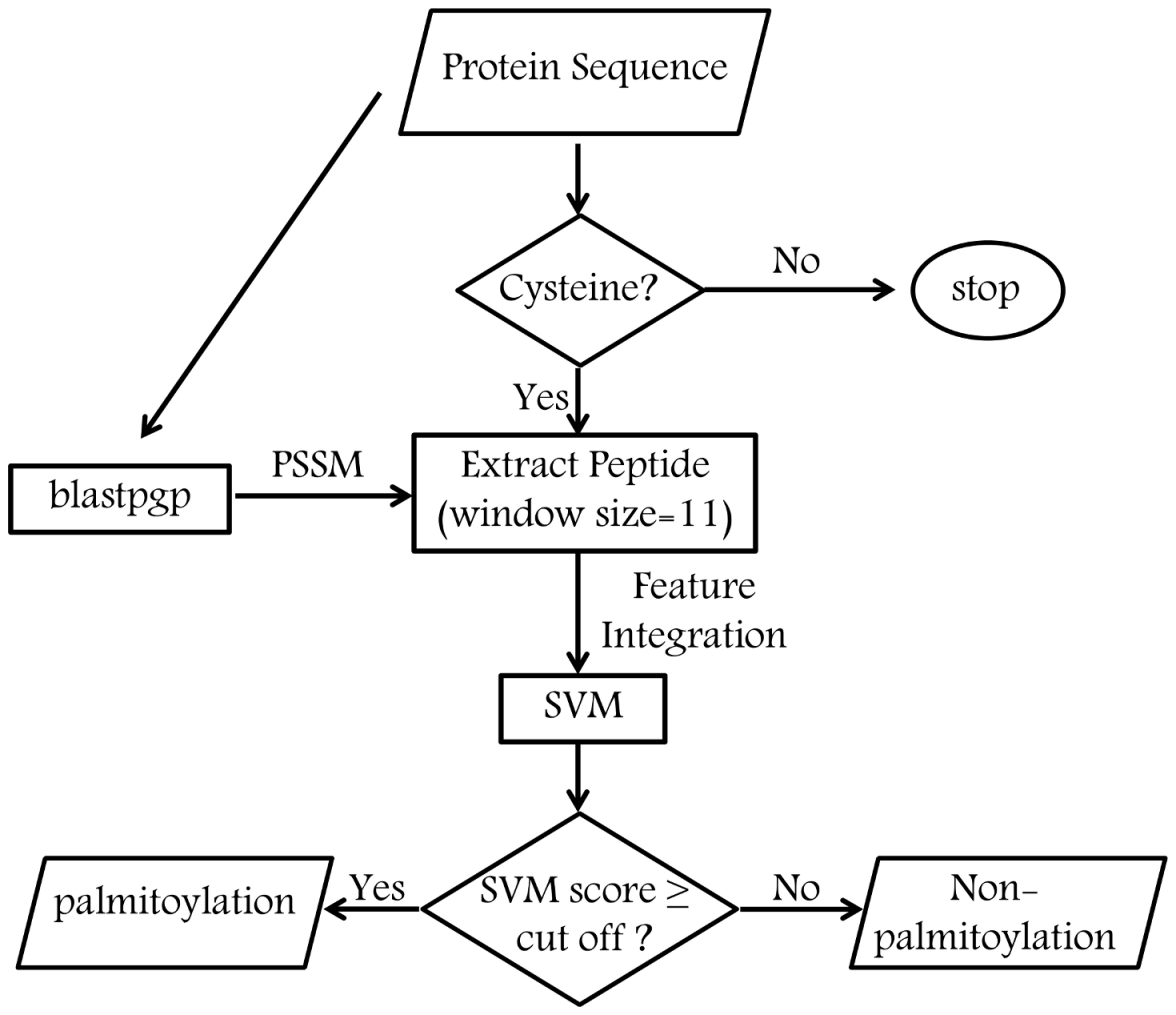
The basic architecture of PalmPred.

In the present study we have described a novel machine learning tool called PalmPred to identify protein palmitoylation sites by using sequence conservation features. LOOCV and benchmarking results showed that PalmPred performed better than the other existing methods. Thus we hope PalmPred may serve as a useful tool to find potential palmitoylation sites in a protein. One downside of our approach is that it takes comparatively more time to generate evolutionary profile however we tried to resolve this issue up to a certain extent by evaluating the performance of PalmPred on less redundant data. The web-interface and standalone of PalmPred is available at http://14.139.227.92/mkumar/palmpred/. The overall working schema for PalmPred is shown in Figure 2.

## Supporting Information

Table S1 **Performance assessment of SVM model based on different databases.**
(DOC)
