# Supplementary material for: PalmPred: An SVM Based Palmitoylation Prediction Method Using Sequence Profile Information
Source: PLoS One. 2014 Feb 19;9(2):e89246. doi: 10.1371/journal.pone.0089246 (PMC3929663; doi:10.1371/journal.pone.0089246)
Supplement: Table S1 — Performance assessment of SVM model based on different databases. (DOC) [file pone.0089246.s001.doc]

**Supporting Information**

**Table S1. Performance assessment of SVM model based on different databases.**

| **Database** | **Sensitivity** | **Specificity** | **Accuracy** | **MCC** |
| --- | --- | --- | --- | --- |
| NR90 | 96.30 | 98.77 | 98.42 | 0.94 |
| NR80 | 92.59 | 98.77 | 97.89 | 0.91 |
| NR70 | 92.59 | 97.55 | 96.84 | 0.88 |
